# The Roles of Indian Hedgehog Signaling in TMJ Formation

**DOI:** 10.3390/ijms20246300

**Published:** 2019-12-13

**Authors:** Till E. Bechtold, Naito Kurio, Hyun-Duck Nah, Cheri Saunders, Paul C. Billings, Eiki Koyama

**Affiliations:** 1 Division of Orthopaedic Surgery, Department of Surgery, The Children’s Hospital of Philadelphia, Philadelphia, PA 19104, USA; tilledward@hotmail.com (T.E.B.); SaundersC1@email.chop.edu (C.S.); BillingsP@email.chop.edu (P.C.B.); 2 Department of Orthodontics, Dentofacial Orthopedics and Pedodontics, Center for Dental and Craniofacial Sciences (CC3), Charité–University Hospital Berlin, D-14197 Berlin, Germany; 3 Division of Plastic and Reconstructive Surgery, Department of Surgery, The Children’s Hospital of Philadelphia, Philadelphia, PA 19104, USA; kurio.naito@tokushima-u.ac.jp (N.K.); NAH@email.chop.edu (H.-D.N.)

**Keywords:** TMJ, synovial joint, articular disc, *Ihh*, *PTHrP*, osteoarthritis

## Abstract

The temporomandibular joint (TMJ) is an intricate structure composed of the mandibular condyle, articular disc, and glenoid fossa in the temporal bone. Apical condylar cartilage is classified as a secondary cartilage, is fibrocartilaginous in nature, and is structurally distinct from growth plate and articular cartilage in long bones. Condylar cartilage is organized in distinct cellular layers that include a superficial layer that produces lubricants, a polymorphic/progenitor layer that contains stem/progenitor cells, and underlying layers of flattened and hypertrophic chondrocytes. Uniquely, progenitor cells reside near the articular surface, proliferate, undergo chondrogenesis, and mature into hypertrophic chondrocytes. During the past decades, there has been a growing interest in the molecular mechanisms by which the TMJ develops and acquires its unique structural and functional features. Indian hedgehog (Ihh), which regulates skeletal development including synovial joint formation, also plays pivotal roles in TMJ development and postnatal maintenance. This review provides a description of the many important recent advances in Hedgehog (Hh) signaling in TMJ biology. These include studies that used conventional approaches and those that analyzed the phenotype of tissue-specific mouse mutants lacking Ihh or associated molecules. The recent advances in understanding the molecular mechanism regulating TMJ development are impressive and these findings will have major implications for future translational medicine tools to repair and regenerate TMJ congenital anomalies and acquired diseases, such as degenerative damage in TMJ osteoarthritic conditions.

## 1. Introduction

**Figure 1:**
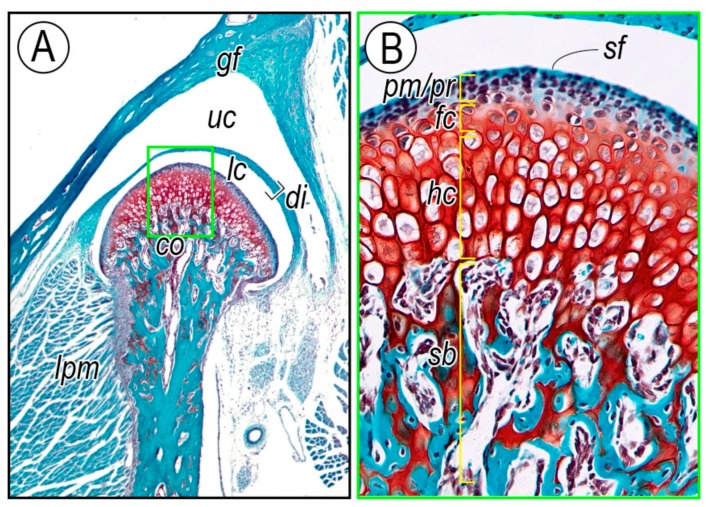
Histology of the TMJ. (**A**) TMJs from 3-month-old wild-type mice were sectioned along their longitudinal axis and sections were stained with safranin O/fast green. (**B**) High-magnification picture of the green boxed area in (**A**), showing the characteristic cellular organization of the condylar cartilage with superficial layer (*sf*), polymorphic/progenitor layer (*pm/pr*), flattened chondrocyte zone (*fc*), hypertrophic chondrocyte zone (*hc*), and subchondral bone (*sb*). *gf*, glenoid fossa; *uc*, upper joint cavity; *di*, articular disc; *lc*, lower joint cavity; *cd*, condyle; *lpm*, lateral pterygoid muscle.

The temporomandibular joint (TMJ), like joints in the shoulder, hip, and knee, is a highly specialized synovial joint and plays a pivotal role in the functioning of the mammalian jaw [1,2,3,4,5]. The TMJ consists of the glenoid fossa in the temporal bone, a condylar head of the mandible, and a fibrocartilaginous articular disc intervening between the fossa and condyle (Figure 1A). Condylar cartilage, unlike the cartilage present in developing limbs, is classified as secondary cartilage, undergoes endochondral ossification, and displays characteristic developmental and growth processes [6,7]. In mammalian embryos, the first overt sign of mandibular condylar development is the appearance of a neural crest-derived cell condensation at the supra-lateral site of the jaw anlagen. The condensation is likely of the periosteal origin within the jaw anlagen [8] or may derive from a separate distinct condensation [9,10]. At this early stage, there is no obvious sign of an intervening articular disc primordium. The condylar condensation differentiates into cartilage and forms a growth plate-like structure, which displays the characteristic zonal organization, consisting of fibroblasts, chondroprogenitor cells, and chondrocytes, along its main axis. These layers are characterized by differences in cell shape and properties and are designated (from the surface): (1) superficial cell layer, (2) fibrous/polymorphic progenitor cell layer, (3) zone of flattened chondrocytes, and (4) zone of hypertrophic chondrocytes (Figure 1B) [11,12,13].

In embryos, the condyle undergoes rapid growth and elongation toward the differentiating temporal bone. Interestingly, the longitudinal growth of the condyle during embryonic and postnatal life primarily results from appositional growth at its apical end, where chondro-progenitor cells residing in the polymorphic cell layer proliferate and differentiate into chondrocytes that in turn become incorporated into the underlying condylar cartilage. Hence, condylar cartilage functions as a growth site of the developing mandibular bone. Therefore, condyle elongation differs from that taking place in other developing skeletal elements, such as long bones or cranial base synchondroses, in which elongation is contributed to by mitotic activity of chondrocytes within the growth plates. With time, the newly differentiated condylar chondrocytes undergo maturation and hypertrophy and are eventually replaced by endochondral bone connecting to the condylar process [14,15,16,17]. A recent study indicated that a small number of chondrocytes may directly differentiate into osteoblasts and form the underlying subarticular bone of the condyle [18].

The development of the articular disc initiates with the formation of a separate flat-shaped ecto-mesenchymal cell condensation located between the developing condylar apex and the glenoid fossa of the temporal bone [19]. With time, the articular disc primordium becomes apparent by the creation of upper and lower articular cavities filled with synovial fluid. The disc subsequently develops into a fibrocartilage structure displaying (1) a biconcave shape with thicker peripheral portions (designated as anterior and posterior bands, respectively) and attaching to the TMJ capsules or the lateral pterygoid muscle and (2) a relatively thin central portion-intermediate zone [19,20]. The TMJ disc and joint cavities enable the condyle to rotate and translate along the glenoid fossa and eminence of the temporal bone during TMJ function.

Although the general development of condyles and articular discs in the TMJ is well understood, comparatively little is known regarding the molecular mechanisms controlling glenoid fossa formation. The glenoid fossa of the temporal bone derives from cranial neural crest cells [4,21,22]. Compared to the articular surface of the mandibular condyle, the articular surface of the glenoid fossa is quite distinct: sporadically distributed chondrocyte progenitors display less proliferative activity and hypertrophic chondrocyte synthesize very little, if any, cartilage matrix [23]. As taking place in developing condylar cartilage, chondrocytes differentiate amongst type I collagen (*Col I*)-expressing mesenchymal cells in a presumptive articulating layer covering the temporal bone where the condyle articulates, exhibiting features of secondary cartilage-like condylar cartilage [24,25]. With time, these chondrocytes undergo endochondral ossification, become entrapped in the intramembranous bony matrix of the temporal bone, and form chondroid bone. Absence or dislocation of the condyle results in arrested glenoid fossa development, suggesting that proper signals and/or mechanical stimulation by the condyle are required to sustain proper glenoid fossa development [26].

Indian hedgehog (Ihh), a member of the Hh family of signaling molecules, is widely recognized as a critical regulator of skeletal development [27,28,29,30]. Ihh is expressed in prehypertrophic and early hypertrophic chondrocytes of the developing growth plate [31,32] and regulates a number of processes including (1) intramembranous bone collar formation [32,33,34], (2) chondrocyte proliferation and maturation rate [35], (3) expression of parathyroid hormone-related protein (PTHrP) in periarticular tissue [36], and (4) endochondral ossification [37,38]. Binding of Ihh to Patched1 (Ptch1), its 12-pass transmembrane receptor, leads to the displacement of Ptch1 from primary cilium, an organelle that bulges from the cell surface. This allows Smoothened (Smo), a 7-pass transmembrane receptor, to be phosphorylated and activate glioma-associated oncogene (Gli) proteins, a family of zinc-finger transcription factors that include Gli1, Gli2, and Gli3. In the absence of Hh ligands, Ptch1 localizes at the base of the primary cilia, preventing Smo from activating the cilium [39,40,41,42,43,44,45]. Under these conditions, Gli2 and Gli3 are subjected to proteolytic cleavage to generate C-terminal truncated forms that repress the transcription of Hh target genes [46,47,48], whereas GLi1, due to a lack of the protein kinase A recognition site necessary for phosphorylation and subsequent cleavage, is thought to function exclusively as an activator [49,50,51]. Studies utilizing *Ihh*-null mouse embryos have provided not only conclusive evidence that Ihh plays multiple roles in long bone development, but also regulates synovial joint formation [1,37,52,53]. The digits of *Ihh*-mutant embryos remain uninterrupted, while heterozygous or wild-type littermates displayed obvious joints. Despite the remarkable nature of these observations and their potentially fundamental implications for other joints in the body, it has remained largely obscure, until quite recently, how Ihh regulates TMJ joint formation, growth, and maintenance [4,54].

In this review, we discuss the important findings on the involvement of Hh signaling in TMJ development during embryonic and early postnatal stages as well as in TMJ establishment and maintenance at postnatal life. We also discuss the possible involvement of Hh pathways in osteoarthritic conditions.

## 2. Recent Experimental Findings

### 2.1. Abnormal TMJ Development in Ihh-Null Mice at Embryonic and Early Postnatal Life

**Figure 2:**
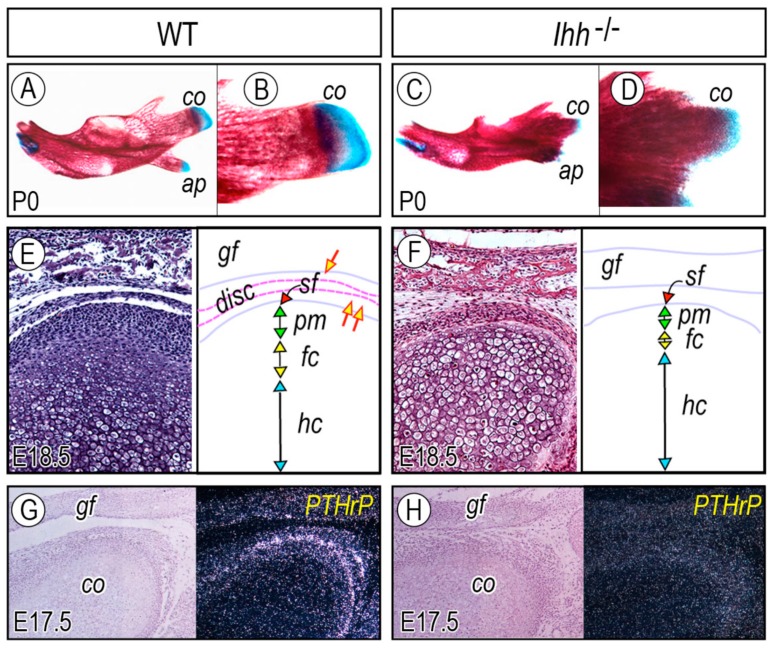
Mandible and TMJ abnormalities in *Ihh*^−/−^ embryos and newborn mice. Mandibles from postnatal day 0 (P0) (**A**–**D**) of (**A**,**B**) wild-type (WT) and (**C**,**D**) *Ihh*^−/−^ skulls were stained with alizarin red and alcian blue. Histological analysis of condylar cartilage from embryonic day 18.5 (E18.5) of (**E**) wild-type and (**F**) *Ihh*^−/−^. Red, green, yellow, and blue vertical lines point to a superficial layer, a polymorphic layer, a flattened chondrocyte layer, and a hypertrophic chondrocyte layer, respectively. Note the absence of the articular disc tissue (disc), the upper joint cavity (arrow), and the lower joint cavity (double arrow). TMJ parasagittal serial sections from E17.5 of (**G**) wild-type (WT) and (**H**) *Ihh*^−/−^ were processed for in situ hybridization with isotope-labeled riboprobe for *PTHrP*. *co*, condyle; *co,* coronoid process; *ap*, angular process; *gf*, glenoid fossa. Figure modified from Shibukawa et al. [11].

It is well established that Indian hedgehog (Ihh) signaling is essential for early axial and appendicular skeletal development [35,36,37,55]. Thus, initial analyses were carried out in skulls from embryonic and newborn (P0) wild-type mice and their corresponding *Ihh*^−/−^ littermates. Wild-type mandibles exhibited their typical elongated morphologies and much of the mandibular body was ossified and stained with alizarin red (Figure 2A). The condylar process (*co*) was prominent and its most apical region contained a typical cartilaginous condyle (Figure 2B). The outer surface of the central and basal regions of the condylar process was surrounded by newly differentiated intramembranous bone (Figure 2B). The angular process (*ap*), where secondary cartilage develops at the apical end, was prominent as well (Figure 2A). In corresponding *Ihh*^−/−^ littermates, the overall length of the mandibular body was reduced as much as 30% and other components, including the condylar process, condyle cartilage, and angular process (*ap*), were all affected (Figure 2C,D).

Detailed histological examination revealed additional structural defects and cellular derangement in *Ihh* mutant TMJs. E15.5 wild-type condyle anlagen contained chondrocytes in their central portions that were circumscribed by a distinct mesenchymal condensation corresponding to disc primordium. By E18.5 to newborn, a complete disc along with upper and lower cavities had formed (Figure 2E, single and double arrows in the right side panel), while condylar chondrocytes displayed typical growth plate-like zonal organization, including a superficial (*sf*) layer, a polymorphic (*pm*)/chondro-progenitor layer, a flattened chondrocyte (*fc*) layer, and hypertrophic chondrocyte (*hc*) layer (Figure 2E). In *Ihh*^−/−^ embryos, condylar chondrocytes were also present by E15.5, but, strikingly, the disc primordium was absent or not discernable. The absence of disc and joint cavities was evident at E18.5 (Figure 2F), such that the condyle directly opposed the glenoid fossa (*gf*). In addition, most of the mutant chondrocytes had undergone hypertrophy by E18.5 with a concurrent reduction in thickness of both the flattened chondrocyte layer (*fc*) and polymorphic (*pm*) layer (Figure 2F). Interestingly, while some phenotypic defects caused by Ihh deficiency are rescued by the concurrent absence of *Gli3* in developing limbs [56], the disc phenotype of *Ihh*^−/−^ mutants was not rescued in double *Ihh*^−/−^*;Gli3*^−/−^ mutants, suggesting unique functions of Ihh in the TMJ [11]. Abnormal formation of the mandibular condyle, articular disc, and joint cavity are also reported in mice carrying mutations in genes (1) directly involved in hedgehog signaling (*Smo*, *Glis*) [57,58], (2) that interact with the hedgehog signaling pathway (*Trps1*) [59], or (3) that reduce or eliminate *Ihh* expression (*Shox2*, *Sox9*) [22,26]. Yang et al. reported that augmented Ihh signaling in cranial neural crest cells caused severe craniofacial abnormalities, including TMJs, where the glenoid fossa was completely absent [60]. Notably, human patients carrying mutations in *Gli2* exhibit a range of facial defects, including mandibular hypoplasia [4,61]. Thus, these observations provide strong evidence that Ihh signaling dictates the cellular organization of the condyle and regulates disc formation and subsequent joint cavitation.

Several lines of evidence indicate that Ihh and PTHrP interact in a negative feedback loop and regulate the onset of chondrocyte hypertrophy in developing long bones [35,36,55]. In the current model, Ihh expressed in prehypertrophic/early hypertrophic chondrocytes signals to the periarticular region and early proliferative chondrocytes at the top of growth plate cartilage to induce PTHrP expression. PTHrP in turn acts on PTHrP receptor-expressing chondrocytes to maintain them in a proliferating and less differentiated state. In developing condylar cartilage, PTHrP is expressed in the superficial and fibrous/chondroprogenitor cells at the apical region of wild-type condylar cartilage by E17.5 (Figure 2G). Importantly, PTHrP expression was drastically reduced or absent in corresponding cell populations in condylar cartilage in *Ihh*^−/−^ embryos (Figure 2H). Given the fact that the number of proliferating chondroprogenitor cells was drastically decreased (ca. 50%) and chondrocytes underwent accelerated hypertrophy in *Ihh*^−/−^ condyles, it is likely that PTHrP induced by Ihh signaling may (1) regulate the proliferation of chondro-progenitor cells and (2) maintain newly differentiated chondrocytes in a less differentiated stage.

Recent studies have suggested that Ihh also acts on chondrocytes to increase rates of proliferation and hypertrophy in a PTHrP-independent manner [62,63]. Expression of PTHrP significantly decreases in the apical region of early postnatal wild-type condyles and is nearly undetectable in juvenile condyles, while chondro-progenitors are still proliferating. Thus, the Ihh-PTHrP feedback loop appears to function primarily during embryogenesis and early postnatal life, while Ihh signaling in juvenile and early adult mice may govern proliferation of chondroprogenitor cells and chondrocyte maturation in a PTHrP-independent manner. Since *Ihh*^−/−^ mice die during embryogenesis or soon after birth, the role(s) that Ihh plays in TMJ growth and maintenance should be investigated using alternative approaches, such as conditional gene knockout techniques employing appropriate inducible Cre mouse lines. Conditional *PTHrP* and compound *PTHrP/Ihh* mutant mice may provide new insights into this important and intriguing area of research. Taken together, studies in embryonic and early postnatal *Ihh*-mutant mice suggest that Ihh is essential for the coordination of (1) intramembranous bone collar formation, (2) progenitor cell proliferation, (3) expression of PTHrP in periarticular tissues, (4) endochondral ossification, and (5) disc and synovial cavity formation.

### 2.2. Role of Ihh in TMJ Growth and Maintenance during Postnatal Stages

#### 2.2.1. Cellular Organization of Condylar Cartilage in Postnatal Stages

**Figure 3:**
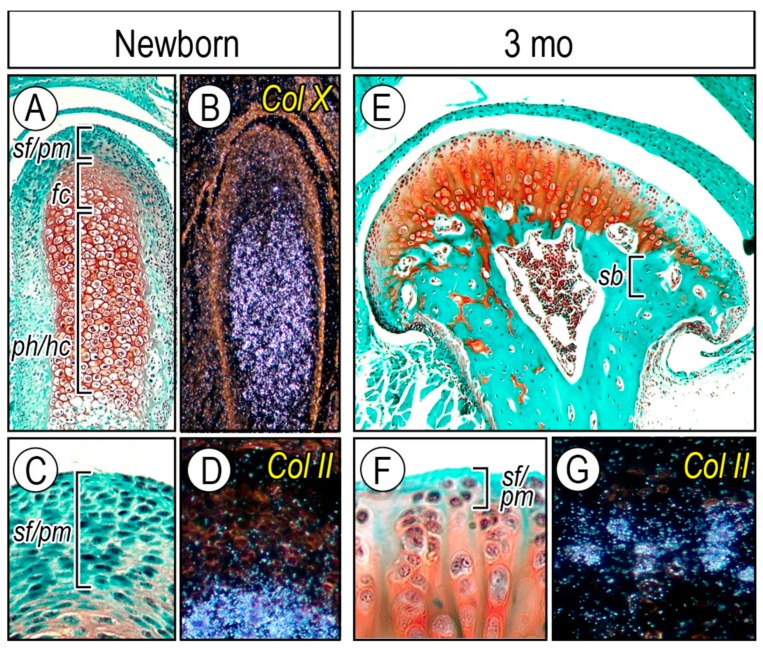
Condylar articular cartilage development and structural organization of a superficial (*sf*) and a polymorphic/progenitor (*pm*/*pr*) layer and chondrocytes with age. Frontal sections from (**A**–**D**) newborn and (**E**–**G**) 3-month-old (3 mo) wild-type mice. (**B**) *Col-X* and (**D**,**G**) *Col-II* gene expression. *sf*, superficial layer; *fc,* flattened chondrocyte layer; *ph/hc,* prehypertrophic and hypertrophic chondrocyte layer; *sb,* subchondral bone. Figure modified from Kurio et al. [67].

The apical layer in developing and adult condyles contains superficial cells producing Proteoglycan 4 (Prg4) and polymorphic cells that display stem cell-like characteristics [64]. Polymorphic cells give rise to chondrocytes for condylar growth and play a role in homeostasis and/or remodeling of condylar cartilage in response to mechanical stress [65,66]. Condylar cartilage length along its longitudinal axis in mice was ca. 470 μm at newborn stages and decreased to ca. 120 μm by 1 month, a thickness maintained through adulthood (Figure 3A,E). Condylar head width along the mediolateral axis was about 150 μm at newborn stages, increased to about 500 μm by 1 month, and remained so thereafter (Figure 3A,E). Subchondral bone plate (*sb*) was fully formed by 3 months of age, supporting articular cartilage (Figure 3E, bracket). Histomorphometric and in situ hybridization analyses revealed that the superficial/polymorphic (*sf*/*pm*) layers positive for fast green staining and less so for Safranin-O were characterized by the lack of type II collagen (*Col-II*) expression (Figure 3C,D). The thickness of superficial/polymorphic layers was ca. 50 μm at the newborn stage, became almost 3 times thinner (ca. 15 µm) by 3 months, and remained so thereafter (Figure 3F,G). Clearly, development, growth, and homeostasis of condylar cartilage during postnatal stages involve a dynamic structural organization of the apical layer (superficial and polymorphic-progenitor layers), whereby chondro-progenitor cells and their progeny cells provide newly differentiated chondrocytes to condylar articular cartilage.

#### 2.2.2. Topography of Hedgehog Signaling

**Figure 4:**
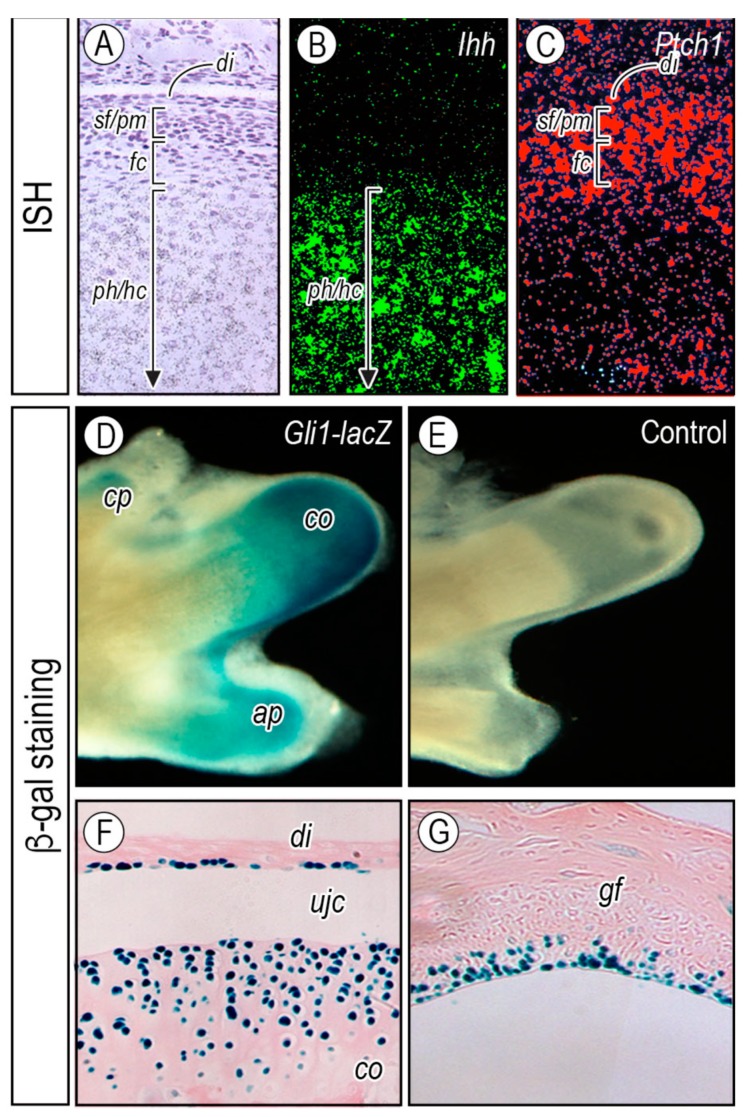
*Ihh*-expressing chondrocytes and its target cells depicted by *Ptch1* expression and *LacZ*-positive cells in postnatal *Gli1*-*nLacZ*-reporter mice. Parasagittal sections from newborn (P1) mice (**A**–**C**) were processed for in situ hybridization with radioisotope-labeled RNA probes of (**B**) *Ihh* and (**C**) *Ptch1*. Whole mount *LacZ* staining of mandible of (**D**) *Gli1*-*nLacZ*-reporter and (**E**) wild-type mice. Histological analyses of (**F**) *lacZ*-stained condyle and (**G**) glenoid fossa of 8-week-old *Gli1*-*nLacZ*-reporter mice. *gf*, glenoid fossa; *di*, articular disc; *ujc*, upper joint cavity; *sf*, superficial layer; *pr*, progenitor layer; *fc,* flattened chondrocyte layer; *ph/hc,* perhypertrophic*/*hypertrophic chondrocyte layer; *cp,* coronoid process*; co,* condyle*; ap*, angular process. Figure modified from Ochiai et al. [69].

Expression of Hh target genes in the condylar cartilage has been investigated to determine whether Hh signaling acts directly or indirectly on joint formation and maintenance in postnatal mice. *Ihh* transcripts were restricted to the prehypertrophic and early hypertrophic chondrocytes (Figure 4A,B). Interestingly, expression of *Ptch1*, a hedgehog receptor and transcriptional target, exhibited a gradient of expression, with relatively low expression levels in the central chondrocyte area of the condyle and higher levels toward the flattened chondrocyte (*fc*), polymorphic (*pm*), and superficial (*sf*) layers and articular disc (*di*) (Figure 4A,C). To determine the actual range of Ihh bioactivity, heterozygous *Gli1*-*nLacZ* embryos, widely used as a functional readout of hedgehog signaling activity, were investigated [67,68]. β-galactosidase activity was detectable over much of the growing condylar cartilage (*co*), the coronoid process (*cp*), and the angular process (*ap*) (Figure 4D) in postnatal day 1 (P1) condyles after processing for whole mount β-galactosidase staining, but was stronger over the entire apical layer of condyles and glenoid fossa (Figure 4F,G) in 8-week-old mice. β-galactosidase activity was also detected in cells lining the disc, with a tendency of β-galactosidase-positive lining cells being more abundant in those facing the lower, rather than upper, joint cavity (Figure 4F). The significance of hedgehog signaling maintained in the postnatal disc cells and the glenoid fossa apical cells needs to be elucidated. These studies indicate that Ihh signaling is active in condylar chondro-progenitors, superficial cells, and disc cells even postnatally, and is likely to influence those cells through life.

#### 2.2.3. Effect of Conditional Ihh Signaling Ablation in Postnatal Stages

**Figure 5:**
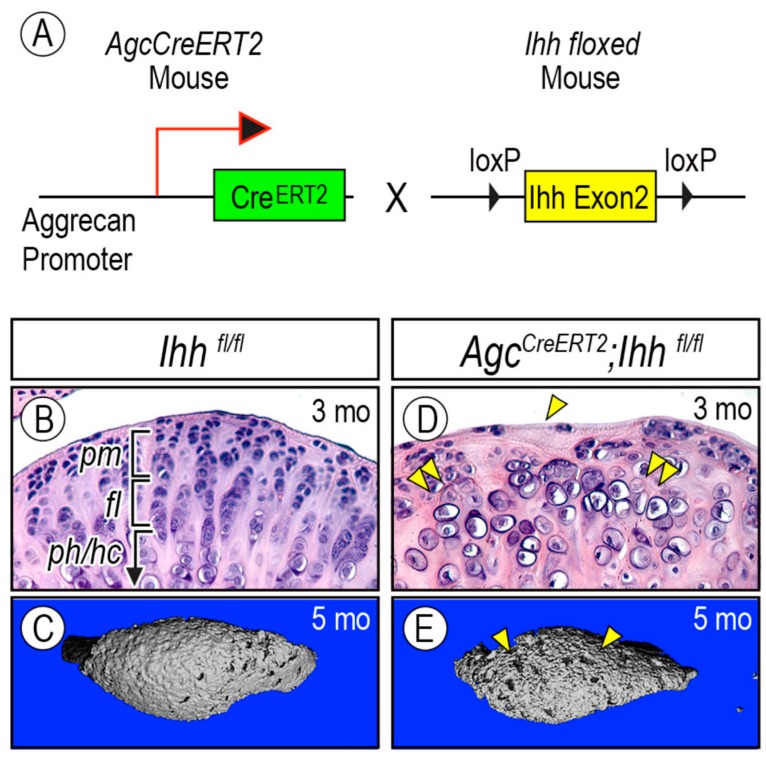
Condylar articular cartilage zonal organization and cellularity are abnormal in *AgcCreER;Ihh^f/f^* mice over time. (**A**) Schematic showing the inducible Cre-Lox system where the floxed-Ihh gene is removed from chondrocytes that express Cre recombinase (arrow). Mice received multiple tamoxifen injections at P14, P21, and P28. TMJs from (**B**,**D**) 3-month-old and (**C**,**E**) 5-month-old of (**B**,**C**) control (*Ihh^f/f^*) mice and (**D**,**E**) *AgcCreER;Ihh^f/f^* mice were analyzed by (**B**,**D**) hematoxylin and eosin staining and (**C**,**E**) µCT. Note the decreased superficial cell number (arrowhead) and the presence of ectopic hypertrophic chondrocytes closer to the condylar surface (double arrowhead) in (**D**). Note that subchondral bone is irregular and porous (arrowheads) in (**E**). *pr*, polymorphic/progenitor layer; *fl*, flattened chondrocyte layer; *hl*, hypertrophic chondrocyte layer. Figure modified from Kurio et al. [67].

As noted above, expression of Hh target genes and β-galactosidase activity in hedgehog reporter mice indicated that an Ihh signaling gradient across the condylar cartilage may contribute to cell function in the progenitor layer and zonal organization in postnatal condylar cartilage. Genetic studies in mice have provided experimental evidence for the significance of Ihh signaling in postnatal TMJ maintenance. To ablate *Ihh* expression in condylar cartilage in the postnatal period, an Aggrecan (*Agc*) *CreER* mouse line was employed [70] (Figure 5A). Compound *Ihh^f/f^;Agc*-*CreER;Gli1*-*nLacZ*, and control (*Ihh^f/f^;Gli1*-*nLacZ*) mice received tamoxifen injections at P14, P21 and P28, and Cre-mediated recombination and subsequent inactivation of Ihh signaling were confirmed by a significant decrease of *Gli1*-*nLacZ*-positive cells in the condylar cartilage. Mutant condylar cartilage displayed decreased numbers of superficial cells and proliferating chondro-progenitor cells and ectopic chondrocyte hypertrophy observed near the articular surface by 3 months old (Figure 5D, arrowhead and double arrowhead, respectively). By 5 months old, μCT analysis revealed that mutant subchondral bone became porous (Figure 5E), leading to decreased bone volume fraction and increased trabecular spacing compared to age-matched controls (Figure 5C). It is likely that decreased Hh signaling is associated with age-related TMJ degenerative changes [66,71]. In senescence-accelerated-prone 8 (SAMP8) mice, which develop early osteoarthritis-like changes in synovial joints at a high frequency [72], condylar cartilage in young SAMP8 mice displayed early-onset degenerative changes, concomitant with reductions in superficial/chondro-progenitor cells, proteoglycan/collagen content, and *Ihh*-expressing chondrocytes [66]. These data clearly demonstrate that Ihh signaling is essential for condylar superficial/progenitor cell layer development and function in postnatal condylar cartilage of TMJs, and its ablation and/or decreased expression in juvenile mice leads to degenerative changes in TMJ condyles, manifesting abnormal chondrocyte maturation and subchondral bone formation in the condyle.

#### 2.2.4. Hh Signaling in Degenerative TMJs

Osteoarthritis (OA) is characterized by the chronic degeneration of various hard and soft tissues around the affected joints. Stress bearing joints of the body, such as the hip and knee, are most commonly affected, but the TMJ is affected as well. TMJ osteoarthritis alters the condylar and glenoid fossa cartilage, subchondral bone, articular disc, and the synovial membrane that, in turn, cause pain and dysfunctional jaw movement [12,73,74,75,76]. There are several contributing factors to TMJ OA inception and progression, including parafunction, occlusion, psychosocial aspects, trauma, and genetics.

Recent studies indicate that decreased lubrication is also associated with the initiation and progression of OA in patients as well as in rodent models after anterior cruciate ligament injury [77,78,79,80]. Lubricin, a mucinous glycoprotein encoded by the proteoglycan 4 (*Prg4*) gene and a major component of synovial fluid, functions as both boundary lubrication and a chondro-protective agent in synovial joints [81,82]. Patients with camptodactyly-arthropathy-coxa vara-pericarditis (CACP) fail to express *PRG4* and subsequently develop polyarthropathy [83,84]. *Prg4*-mutant mice develop OA-like phenotypes in synovial joints, implying that Prg4 may have important roles in joint maintenance [85,86]. While TMJs in *Prg4*^−/−^ mice developed normally, mutant mice developed degenerative changes. *Prg4*-mutant mice exhibited hyperplasia in the glenoid fossa articular cartilage, articular disc, and synovial membrane as early as 2 weeks of age and osteoarthritic changes in articular cartilage of the glenoid fossa and condyle by 6 months, in which loss of proteoglycans, an increase in osteoclast activity, and subchondral bone loss were observed [24,87]. Interestingly, these degenerative changes occurred earlier and were more severe than those in knee and hip joints, indicating that TMJs are more vulnerable to the loss of lubricin than other joints [87]. It has been reported that compound mutants of biglycan and fibromodulin, members of the small leucine-rich repeat proteoglycan family, display OA-like phenotypes in the knee joints much earlier than in TMJ [88,89,90]. Thus, these results suggest that synovial fluid plays an important role(s) in TMJ function and maintenance.

**Figure 6:**
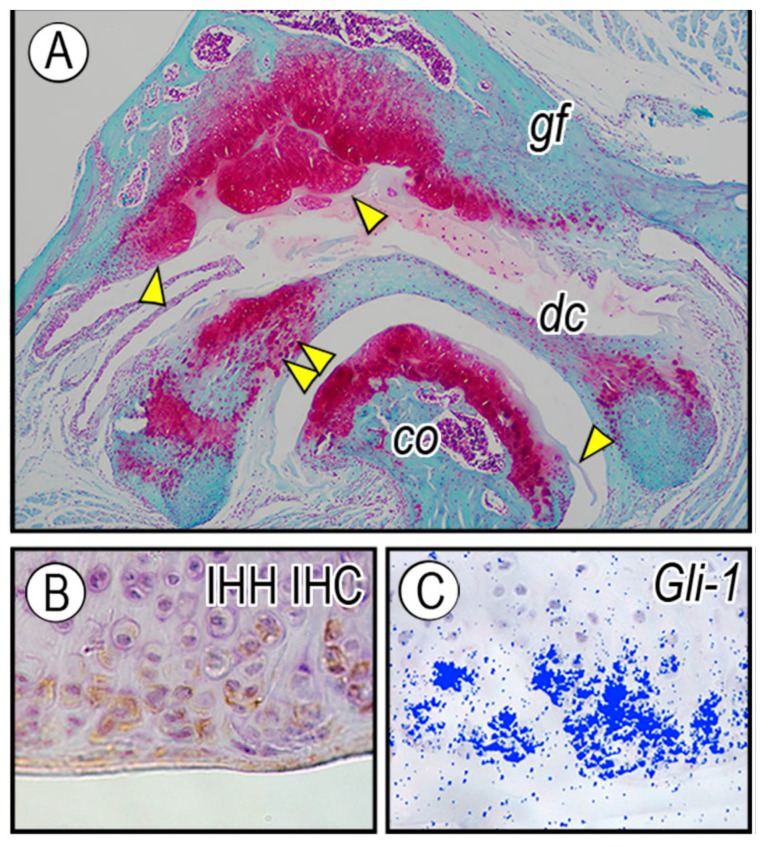
Ectopic expression of *Ihh* in osteophyte-developing glenoid fossa cartilage in *Prg4*-null mice. TMJs from (**A**) 15-month-old *Prg4*^−/−^ mice were analyzed by Safranin O/fast green staining. Note that osteophytes are developing from the glenoid fossa, along with condylar cartilage (arrowhead) and ectopic cartilage formation in disc (double arrowhead). (**B**) Immunohistochemistry (IHC) with IHH antibody and (**C**) in situ hybridization of *Gli-1* mRNA in the developing osteophytes. Figure modified from Bechtold et al. [25].

Osteophyte, a fibrocartilage-capped bonny outgrowth, is a hallmark radiographic feature of degenerative TMJ joint disease [91]. Joint instability likely contributes to osteophyte formation in the articular surface of the condyle and glenoid fossa. Compared to the development of OA in synovial joints of appendicular skeletal elements, the prevalence of osteophyte formation in TMJ OA is relatively rare. However, once developed, it causes various clinical symptoms and subsequently compromises joint function [92]. While Transforming growth factor β (TGFβ), Bone morphogenetic proteins (BMPs), Fibroblast growth factors (FGFs), or insulin-like growth factor-1 (IGF-1) have been detected in the developing osteophyte [93,94,95,96,97], what causes osteophytes in TMJs remains obscure. Interestingly, *Prg4*^−/−^ mice exhibit increased osteophyte formation in the condylar cartilage and glenoid fossa with age (Figure 6A, arrowhead) [25,87,98]. This study showed that expression levels of *Ihh*, *Gli-1, Sox9*, and *Aggrecan* (*Agc*) (the latter 2 genes are markers of chondro-progenitors and chondrocytes, respectively) increased in osteophytes developing in the affected glenoid fossa. Immunohistochemistry revealed that IHH was preferentially distributed in the peripheral cells of osteophytes and underlying chondrocytes (Figure 6B). *Gli-1* transcripts were expressed in cells residing at the apical region of developing osteophytes (Figure 6C), indicative of Hh signaling activation as well as chondrogenesis taking place at this site. Expression of *PTHrP* and its receptor *Pth1r* was increased in *Prg4*^−/−^ glenoid fossa. In glenoid fossa cells in culture, Hh signaling stimulated chondrocyte differentiation and maturation, evaluated by increased chondrocyte proteoglycan synthesis and alkaline phosphatase activity, respectively, while treatment with hedgehog inhibitor, Hh Antag, prevented such maturation process [25]. In line with these results, data with *Col2*-*CreER;Pth1r^fl/fl^;Smo^fl/fl^* mice suggest that inhibition of Ihh signaling in osteoarthritis-like TMJs prevents chondrocyte terminal differentiation through a Pth1r-dependent mechanism [99]. Further studies are warranted to determine the pathophysiology underlying activation of Ihh and PTHrP signaling in osteoarthritic TMJs.

## 3. Perspectives

While a number of studies have addressed the importance of the Hh signaling pathway in TMJ biology, there are many questions that remain unanswered.

First, data summarized in this review show long-range signaling of Ihh proteins during embryonic development and postnatal growth. However, the underlying molecular mechanisms regulating Ihh protein release from the cell surface need to be further clarified. Multiple studies indicate that such long-range signaling of hedgehog requires lipid modifications that promote the formation of multimeric complexes, the formation of which depends on the palmitoylation and addition of cholesterol to the N-terminal hedgehog fragments [100,101,102]. Heparan sulfate proteoglycans (HSPGs) with which hedgehog proteins interact through their Cardin–Weintraub motif, could allow formation of hedgehog multimers, facilitating Hh protein oligomerization [103,104,105,106,107]. Following oligomerization, Hh proteins bind the membrane protein Dispatched (Disp) in a cholesterol-dependent manner and the combined action of Disp and Scube2, a secreted protein, release oligomerized Hh proteins from the cells [108,109,110,111]. Studies suggest that the Golgi-associated *N*-sulfotransferase 1(Ndst-1), which catalyzes the sulfation of HSPG glycosaminoglycan chains, is critical for organogenesis [112,113,114], including mandibular condyles and TMJ development, and allows HSPGs to exert their roles via regulation of Ihh signaling topography and action [112,113,114].

Second, there is a critical need for in vivo and in vitro studies to further define interactions between Ihh and other signaling pathways that regulate postnatal morphogenesis and growth of TMJs. In mouse embryos, Ihh signaling promotes expression of PTHrP at the apical end of the presumptive condylar cartilage, which leads to increased numbers of presumptive chondro-progenitors [11,35,36,37]. Notably, the size of the condylar cartilage in young adult mice, the length along anteroposterior and mediolateral axes, are about 3 times larger compared with prenatal mice [67]. As indicated above, the expression of PTHrP is high during embryogenesis and early postnatal life, but declines in juvenile mice. Thus, further studies are required to define the role of Ihh signaling during the growth and development of the TMJ and associated tissues in the presence and absence of PTHrP.

Third, the role(s) that altered Ihh signaling and associated pathways, such as primary cilia components, play in the degenerative changes that accompany osteoarthritis are not fully understood in the TMJ or synovial joints [25,69,98,99,115,116]. For example, it has been observed that activation of Hh signaling leads to the induction of ectopic chondrocyte hypertrophy in degenerative articular cartilage [28,115,117]. Further, numerous studies have demonstrated that several signaling pathways, including TGFβ, BMP, IGF-1, and FGF, are up-regulated during osteophyte formation in synovial joints [93,94,95,96,97]. Interestingly, altered Hh and *PTHrP* signaling has been detected in osteophytes developing at the surface of the glenoid fossa and condylar cartilage [25].

While there has been much progress defining the roles that different signaling pathways play during normal TMJ growth and development and the pathological changes giving rise to osteoarthritis, much remains to explored. Future studies need to define the interactions between multiple signaling pathways and determine how this ‘crosstalk’ directs TMJ morphogenesis and more broadly, bone and cartilage differentiation. The results from these studies will provide a solid basis leading to the development of new and novel approaches for repairing TMJ congenital anomalies and acquired degenerative damage resulting from osteoarthritic conditions.

## References

[1] Rux D., Decker R.S., Koyama E., Pacifici M. (2019). Joints in the appendicular skeleton: Developmental mechanisms and evolutionary influences. Curr. Top. Dev. Biol..

[2] Pacifici M., Decker R.S., Koyama E. (2018). Limb Synovial Joint Development From the Hips Down: Implications for Articular Cartilage Repair and Regeneration. Developmental Biology and Musculoskeletal Tissue Engineering.

[3] Koyama E., Yasuda T., Minugh-Purvis N., Kinumatsu T., Yallowitz A.R., Wellik D.M., Pacifici M. (2010). Hox11 genes establish synovial joint organization and phylogenetic characteristics in developing mouse zeugopod skeletal elements. Development.

[4] Hinton R.J. (2014). Genes that regulate morphogenesis and growth of the temporomandibular joint: A review. Dev. Dyn..

[5] Suzuki A., Iwata J. (2016). Mouse genetic models for temporomandibular joint development and disorders. Oral Dis..

[6] Symons N.B. (1965). A histochemical study of the secondary cartilage of the mandibular condyle in the rat. Arch. Oral Biol..

[7] Koyama E., Shibukawa Y., Nagayama M., Sugito H., Young B., Yuasa T., Okabe T., Ochiai T., Kamiya N., Rountree R.B. (2008). A distinct cohort of progenitor cells participates in synovial joint and articular cartilage formation during mouse limb skeletogenesis. Dev. Biol..

[8] Shibata S., Fukada K., Suzuki S., Ogawa T., Yamashita Y. (2002). In situ hybridization and immunohistochemistry of bone sialoprotein and secreted phosphoprotein 1 (osteopontin) in the developing mouse mandibular condylar cartilage compared with limb bud cartilage. J. Anat..

[9] Vinkka H. (1982). Secondary cartilages in the facial skeleton of the rat. Proc. Finn. Dent. Soc..

[10] Radlanski R.J., Renz H., Klarkowski M.C. (2003). Prenatal development of the human mandible. 3D reconstructions, morphometry and bone remodelling pattern, sizes 12-117 mm CRL. Anat. Embryol..

[11] Shibukawa Y., Young B., Wu C., Yamada S., Long F., Pacifici M., Koyama E. (2007). Temporomandibular joint formation and condyle growth require Indian hedgehog signaling. Dev. Dyn..

[12] Wadhwa S., Kapila S. (2008). TMJ disorders: Future innovations in diagnostics and therapeutics. J. Dent. Educ..

[13] Luder H.U., Leblond C.P., von der Mark K. (1988). Cellular stages in cartilage formation as revealed by morphometry, radioautography and type II collagen immunostaining of the mandibular condyle from weanling rats. Am. J. Anat.

[14] Silbermann M., Frommer J. (1972). The nature of endochondral ossification in the mandibular condyle of the mouse. Anat. Rec..

[15] Sarnat B.G. (1966). Developmental facial abnormalities and the temporomandibular joint. Dent. Clin. N. Am..

[16] Petrovic A.G. (1972). Mechanisms and regulation of mandibular condylar growth. Acta Morphol. Neerl. Scand..

[17] Kantomaa T., Tuominen M., Pirttiniemi P. (1994). Effect of mechanical forces on chondrocyte maturation and differentiation in the mandibular condyle of the rat. J. Dent. Res..

[18] Jing Y., Zhou X., Han X., Jing J., von der Mark K., Wang J., de Crombrugghe B., Hinton R.J., Feng J.Q. (2015). Chondrocytes Directly Transform into Bone Cells in Mandibular Condyle Growth. J. Dent. Res..

[19] Frommer J. (1964). Prenatal Development of the Mandibular Joint in Mice. Anat. Rec..

[20] Bhaskar S.N. (1953). Growth pattern of the rat mandible from 13 days insemination age to 30 days after birth. Am. J. Anat.

[21] Mori-Akiyama Y., Akiyama H., Rowitch D.H., de Crombrugghe B. (2003). Sox9 is required for determination of the chondrogenic cell lineage in the cranial neural crest. Proc. Natl. Acad. Sci. USA.

[22] Gu S., Wei N., Yu L., Fei J., Chen Y. (2008). Shox2-deficiency leads to dysplasia and ankylosis of the temporomandibular joint in mice. Mech. Dev..

[23] Yasuda T., Nah H.D., Laurita J., Kinumatsu T., Shibukawa Y., Shibutani T., Minugh-Purvis N., Pacifici M., Koyama E. (2012). Muenke syndrome mutation, FgfR3P(2)(4)(4)R, causes TMJ defects. J. Dent. Res..

[24] Koyama E., Saunders C., Salhab I., Decker R.S., Chen I., Um H., Pacifici M., Nah H.D. (2014). Lubricin is Required for the Structural Integrity and Post-natal Maintenance of TMJ. J. Dent. Res..

[25] Bechtold T.E., Saunders C., Decker R.S., Um H.B., Cottingham N., Salhab I., Kurio N., Billings P.C., Pacifici M., Nah H.D. (2016). Osteophyte formation and matrix mineralization in a TMJ osteoarthritis mouse model are associated with ectopic hedgehog signaling. Matrix Biol..

[26] Wang Y., Liu C., Rohr J., Liu H., He F., Yu J., Sun C., Li L., Gu S., Chen Y. (2011). Tissue interaction is required for glenoid fossa development during temporomandibular joint formation. Dev. Dyn..

[27] Lee R.T., Zhao Z., Ingham P.W. (2016). Hedgehog signalling. Development.

[28] Alman B.A. (2015). The role of hedgehog signalling in skeletal health and disease. Nat. Rev. Rheumatol..

[29] Yao E., Chuang P.T. (2015). Hedgehog signaling: From basic research to clinical applications. J. Formos. Med. Assoc..

[30] Abramyan J. (2019). Hedgehog Signaling and Embryonic Craniofacial Disorders. J. Dev. Biol..

[31] Bitgood M.J., McMahon A.P. (1995). Hedgehog and Bmp genes are coexpressed at many diverse sites of cell-cell interaction in the mouse embryo. Dev. Biol..

[32] Koyama E., Leatherman J.L., Noji S., Pacifici M. (1996). Early chick limb cartilaginous elements possess polarizing activity and express hedgehog-related morphogenetic factors. Dev. Dyn..

[33] Nakamura T., Aikawa T., Iwamoto-Enomoto M., Iwamoto M., Higuchi Y., Pacifici M., Kinto N., Yamaguchi A., Noji S., Kurisu K. (1997). Induction of osteogenic differentiation by hedgehog proteins. Biochem. Biophys. Res. Commun..

[34] Long F., Chung U.I., Ohba S., McMahon J., Kronenberg H.M., McMahon A.P. (2004). Ihh signaling is directly required for the osteoblast lineage in the endochondral skeleton. Development.

[35] Vortkamp A., Lee K., Lanske B., Segre G.V., Kronenberg H.M., Tabin C.J. (1996). Regulation of rate of cartilage differentiation by Indian hedgehog and PTH-related protein. Science.

[36] Lanske B., Karaplis A.C., Lee K., Luz A., Vortkamp A., Pirro A., Karperien M., Defize L.H., Ho C., Mulligan R.C. (1996). PTH/PTHrP receptor in early development and Indian hedgehog-regulated bone growth. Science.

[37] St-Jacques B., Hammerschmidt M., McMahon A.P. (1999). Indian hedgehog signaling regulates proliferation and differentiation of chondrocytes and is essential for bone formation. Genes Dev..

[38] Chung U.I., Schipani E., McMahon A.P., Kronenberg H.M. (2001). Indian hedgehog couples chondrogenesis to osteogenesis in endochondral bone development. J. Clin. Investig..

[39] Alcedo J., Ayzenzon M., Von Ohlen T., Noll M., Hooper J.E. (1996). The Drosophila smoothened gene encodes a seven-pass membrane protein, a putative receptor for the hedgehog signal. Cell.

[40] van den Heuvel M., Ingham P.W. (1996). smoothened encodes a receptor-like serpentine protein required for hedgehog signalling. Nature.

[41] Chen W., Burgess S., Hopkins N. (2001). Analysis of the zebrafish smoothened mutant reveals conserved and divergent functions of hedgehog activity. Development.

[42] Goodrich L.V., Milenkovic L., Higgins K.M., Scott M.P. (1997). Altered neural cell fates and medulloblastoma in mouse patched mutants. Science.

[43] Haycraft C.J., Serra R. (2008). Cilia involvement in patterning and maintenance of the skeleton. Curr. Top. Dev. Biol..

[44] Sreekumar V., Norris D.P. (2019). Cilia and development. Curr. Opin. Genet. Dev..

[45] Nachury M.V., Mick D.U. (2019). Establishing and regulating the composition of cilia for signal transduction. Nat. Rev. Mol. Cell Biol..

[46] Ruiz i Altaba A. (1999). Gli proteins encode context-dependent positive and negative functions: Implications for development and disease. Development.

[47] Sasaki H., Nishizaki Y., Hui C., Nakafuku M., Kondoh H. (1999). Regulation of Gli2 and Gli3 activities by an amino-terminal repression domain: Implication of Gli2 and Gli3 as primary mediators of Shh signaling. Development.

[48] Wong S.Y., Reiter J.F. (2008). The primary cilium at the crossroads of mammalian hedgehog signaling. Curr. Top. Dev. Biol..

[49] Hynes M., Stone D.M., Dowd M., Pitts-Meek S., Goddard A., Gurney A., Rosenthal A. (1997). Control of cell pattern in the neural tube by the zinc finger transcription factor and oncogene Gli-1. Neuron.

[50] Karlstrom R.O., Tyurina O.V., Kawakami A., Nishioka N., Talbot W.S., Sasaki H., Schier A.F. (2003). Genetic analysis of zebrafish gli1 and gli2 reveals divergent requirements for gli genes in vertebrate development. Development.

[51] Lee J., Platt K.A., Censullo P., Ruiz i Altaba A. (1997). Gli1 is a target of Sonic hedgehog that induces ventral neural tube development. Development.

[52] Koyama E., Ochiai T., Rountree R.B., Kingsley D.M., Enomoto-Iwamoto M., Iwamoto M., Pacifici M. (2007). Synovial joint formation during mouse limb skeletogenesis: Roles of Indian hedgehog signaling. Ann. N. Y. Acad. Sci..

[53] Decker R.S., Koyama E., Pacifici M. (2014). Genesis and morphogenesis of limb synovial joints and articular cartilage. Matrix Biol..

[54] Kubiak M., Ditzel M. (2016). A Joint Less Ordinary: Intriguing Roles for Hedgehog Signalling in the Development of the Temporomandibular Synovial Joint. J. Dev. Biol..

[55] Maeda Y., Nakamura E., Nguyen M.T., Suva L.J., Swain F.L., Razzaque M.S., Mackem S., Lanske B. (2007). Indian Hedgehog produced by postnatal chondrocytes is essential for maintaining a growth plate and trabecular bone. Proc. Natl. Acad. Sci. USA.

[56] Hilton M.J., Tu X., Cook J., Hu H., Long F. (2005). Ihh controls cartilage development by antagonizing Gli3, but requires additional effectors to regulate osteoblast and vascular development. Development.

[57] Purcell P., Joo B.W., Hu J.K., Tran P.V., Calicchio M.L., O’Connell D.J., Maas R.L., Tabin C.J. (2009). Temporomandibular joint formation requires two distinct hedgehog-dependent steps. Proc. Natl. Acad. Sci. USA.

[58] Mo R., Freer A.M., Zinyk D.L., Crackower M.A., Michaud J., Heng H.H., Chik K.W., Shi X.M., Tsui L.C., Cheng S.H. (1997). Specific and redundant functions of Gli2 and Gli3 zinc finger genes in skeletal patterning and development. Development.

[59] Michikami I., Fukushi T., Honma S., Yoshioka S., Itoh S., Muragaki Y., Kurisu K., Ooshima T., Wakisaka S., Abe M. (2012). Trps1 is necessary for normal temporomandibular joint development. Cell Tissue Res..

[60] Yang L., Gu S., Ye W., Song Y., Chen Y. (2016). Augmented Indian hedgehog signaling in cranial neural crest cells leads to craniofacial abnormalities and dysplastic temporomandibular joint in mice. Cell Tissue Res..

[61] Bertolacini C.D., Ribeiro-Bicudo L.A., Petrin A., Richieri-Costa A., Murray J.C. (2012). Clinical findings in patients with GLI2 mutations—Phenotypic variability. Clin. Genet..

[62] Mak K.K., Kronenberg H.M., Chuang P.T., Mackem S., Yang Y. (2008). Indian hedgehog signals independently of PTHrP to promote chondrocyte hypertrophy. Development.

[63] Karp S.J., Schipani E., St-Jacques B., Hunzelman J., Kronenberg H., McMahon A.P. (2000). Indian hedgehog coordinates endochondral bone growth and morphogenesis via parathyroid hormone related-protein-dependent and -independent pathways. Development.

[64] Embree M.C., Chen M., Pylawka S., Kong D., Iwaoka G.M., Kalajzic I., Yao H., Shi C., Sun D., Sheu T.J. (2016). Exploiting endogenous fibrocartilage stem cells to regenerate cartilage and repair joint injury. Nat. Commun..

[65] Kaul R., O’Brien M.H., Dutra E., Lima A., Utreja A., Yadav S. (2016). The Effect of Altered Loading on Mandibular Condylar Cartilage. PLoS ONE.

[66] Ishizuka Y., Shibukawa Y., Nagayama M., Decker R., Kinumatsu T., Saito A., Pacifici M., Koyama E. (2014). TMJ degeneration in SAMP8 mice is accompanied by deranged Ihh signaling. J. Dent. Res..

[67] Kurio N., Saunders C., Bechtold T.E., Salhab I., Nah H.D., Sinha S., Billings P.C., Pacifici M., Koyama E. (2018). Roles of Ihh signaling in chondroprogenitor function in postnatal condylar cartilage. Matrix Biol..

[68] Bai C.B., Auerbach W., Lee J.S., Stephen D., Joyner A.L. (2002). Gli2, but not Gli1, is required for initial Shh signaling and ectopic activation of the Shh pathway. Development.

[69] Ochiai T., Shibukawa Y., Nagayama M., Mundy C., Yasuda T., Okabe T., Shimono K., Kanyama M., Hasegawa H., Maeda Y. (2010). Indian hedgehog roles in post-natal TMJ development and organization. J. Dent. Res..

[70] Henry S.P., Jang C.W., Deng J.M., Zhang Z., Behringer R.R., de Crombrugghe B. (2009). Generation of aggrecan-CreERT2 knockin mice for inducible Cre activity in adult cartilage. Genesis.

[71] Chen W.H., Hosokawa M., Tsuboyama T., Ono T., Iizuka T., Takeda T. (1989). Age-related changes in the temporomandibular joint of the senescence accelerated mouse. SAM-P/3 as a new murine model of degenerative joint disease. Am. J. Pathol..

[72] Hosokawa M., Kasai R., Higuchi K., Takeshita S., Shimizu K., Hamamoto H., Honma A., Irino M., Toda K., Matsumura A. (1984). Grading score system: A method for evaluation of the degree of senescence in senescence accelerated mouse (SAM). Mech. Ageing Dev..

[73] Scrivani S.J., Keith D.A., Kaban L.B. (2008). Temporomandibular disorders. N. Engl. J. Med..

[74] Dimitroulis G. (2018). Management of temporomandibular joint disorders: A surgeon’s perspective. Aust. Dent. J..

[75] Mercuri L.G. (2008). Osteoarthritis, osteoarthrosis, and idiopathic condylar resorption. Oral Maxillofac. Surg. Clin. N. Am..

[76] Schiffman E., Ohrbach R., Truelove E., Look J., Anderson G., Goulet J.P., List T., Svensson P., Gonzalez Y., Lobbezoo F. (2014). Diagnostic Criteria for Temporomandibular Disorders (DC/TMD) for Clinical and Research Applications: Recommendations of the International RDC/TMD Consortium Network* and Orofacial Pain Special Interest Groupdagger. J. Oral Fac. Pain Headache.

[77] Elsaid K.A., Machan J.T., Waller K., Fleming B.C., Jay G.D. (2009). The impact of anterior cruciate ligament injury on lubricin metabolism and the effect of inhibiting tumor necrosis factor alpha on chondroprotection in an animal model. Arthritis Rheum..

[78] Elsaid K.A., Fleming B.C., Oksendahl H.L., Machan J.T., Fadale P.D., Hulstyn M.J., Shalvoy R., Jay G.D. (2008). Decreased lubricin concentrations and markers of joint inflammation in the synovial fluid of patients with anterior cruciate ligament injury. Arthritis Rheum..

[79] Teeple E., Elsaid K.A., Fleming B.C., Jay G.D., Aslani K., Crisco J.J., Mechrefe A.P. (2008). Coefficients of friction, lubricin, and cartilage damage in the anterior cruciate ligament-deficient guinea pig knee. J. Orthop. Res..

[80] Kosinska M.K., Ludwig T.E., Liebisch G., Zhang R., Siebert H.C., Wilhelm J., Kaesser U., Dettmeyer R.B., Klein H., Ishaque B. (2015). Articular Joint Lubricants during Osteoarthritis and Rheumatoid Arthritis Display Altered Levels and Molecular Species. PLoS ONE.

[81] Jay G.D., Waller K.A. (2014). The biology of lubricin: Near frictionless joint motion. Matrix Biol..

[82] Das N., Schmidt T.A., Krawetz R.J., Dufour A. (2019). Proteoglycan 4: From Mere Lubricant to Regulator of Tissue Homeostasis and Inflammation: Does proteoglycan 4 have the ability to buffer the inflammatory response?. Bioessays.

[83] Bahabri S.A., Suwairi W.M., Laxer R.M., Polinkovsky A., Dalaan A.A., Warman M.L. (1998). The camptodactyly-arthropathy-coxa vara-pericarditis syndrome: Clinical features and genetic mapping to human chromosome 1. Arthritis Rheum..

[84] Marcelino J., Carpten J.D., Suwairi W.M., Gutierrez O.M., Schwartz S., Robbins C., Sood R., Makalowska I., Baxevanis A., Johnstone B. (1999). CACP, encoding a secreted proteoglycan, is mutated in camptodactyly-arthropathy-coxa vara-pericarditis syndrome. Nat. Genet..

[85] Hill A., Waller K.A., Cui Y., Allen J.M., Smits P., Zhang L.X., Ayturk U.M., Hann S., Lessard S.G., Zurakowski D. (2015). Lubricin restoration in a mouse model of congenital deficiency. Arthritis Rheumatol..

[86] Rhee D.K., Marcelino J., Baker M., Gong Y., Smits P., Lefebvre V., Jay G.D., Stewart M., Wang H., Warman M.L. (2005). The secreted glycoprotein lubricin protects cartilage surfaces and inhibits synovial cell overgrowth. J. Clin. Investig..

[87] Hill A., Duran J., Purcell P. (2014). Lubricin protects the temporomandibular joint surfaces from degeneration. PLoS ONE.

[88] Chen J., Gupta T., Barasz J.A., Kalajzic Z., Yeh W.C., Drissi H., Hand A.R., Wadhwa S. (2009). Analysis of microarchitectural changes in a mouse temporomandibular joint osteoarthritis model. Arch. Oral Biol..

[89] Wadhwa S., Embree M., Ameye L., Young M.F. (2005). Mice deficient in biglycan and fibromodulin as a model for temporomandibular joint osteoarthritis. Cells Tissues Organs.

[90] Embree M.C., Kilts T.M., Ono M., Inkson C.A., Syed-Picard F., Karsdal M.A., Oldberg A., Bi Y., Young M.F. (2010). Biglycan and fibromodulin have essential roles in regulating chondrogenesis and extracellular matrix turnover in temporomandibular joint osteoarthritis. Am. J. Pathol..

[91] Larheim T.A., Abrahamsson A.K., Kristensen M., Arvidsson L.Z. (2015). Temporomandibular joint diagnostics using CBCT. Dentomaxillofac. Radiol..

[92] Rehan O.M., Saleh H.A.K., Raffat H.A., Abu-Taleb N.S. (2018). Osseous changes in the temporomandibular joint in rheumatoid arthritis: A cone-beam computed tomography study. Imaging Sci. Dent..

[93] Murata K., Kokubun T., Onitsuka K., Oka Y., Kano T., Morishita Y., Ozone K., Kuwabara N., Nishimoto J., Isho T. (2019). Controlling joint instability after anterior cruciate ligament transection inhibits transforming growth factor-beta-mediated osteophyte formation. Osteoarthr. Cartil..

[94] van der Kraan P.M., van den Berg W.B. (2007). Osteophytes: Relevance and biology. Osteoarthr. Cartil..

[95] Blaney Davidson E.N., Vitters E.L., van der Kraan P.M., van den Berg W.B. (2006). Expression of transforming growth factor-beta (TGFbeta) and the TGFbeta signalling molecule SMAD-2P in spontaneous and instability-induced osteoarthritis: Role in cartilage degradation, chondrogenesis and osteophyte formation. Ann. Rheum. Dis..

[96] Jingushi S., Shida J., Iwamoto Y., Kinoshita T., Hiyama Y., Tamura M., Izumi T. (2006). Transient exposure of fibroblast growth factor-2 induced proliferative but not destructive changes in mouse knee joints. Connect. Tissue Res..

[97] Okazaki K., Jingushi S., Ikenoue T., Urabe K., Sakai H., Ohtsuru A., Akino K., Yamashita S., Nomura S., Iwamoto Y. (1999). Expression of insulin-like growth factor I messenger ribonucleic acid in developing osteophytes in murine experimental osteoarthritis and in rats inoculated with growth hormone-secreting tumor. Endocrinology.

[98] Bechtold T.E., Saunders C., Mundy C., Um H., Decker R.S., Salhab I., Kurio N., Billings P.C., Pacifici M., Nah H.D. (2016). Excess BMP Signaling in Heterotopic Cartilage Forming in Prg4-null TMJ Discs. J. Dent. Res..

[99] Yang H., Zhang M., Liu Q., Zhang H., Zhang J., Lu L., Xie M., Chen D., Wang M. (2019). Inhibition of Ihh Reverses Temporomandibular Joint Osteoarthritis via a PTH1R Signaling Dependent Mechanism. Int. J. Mol. Sci..

[100] Cohen M.M. (2003). The hedgehog signaling network. Am. J. Med. Genet. A.

[101] Pepinsky R.B., Zeng C., Wen D., Rayhorn P., Baker D.P., Williams K.P., Bixler S.A., Ambrose C.M., Garber E.A., Miatkowski K. (1998). Identification of a palmitic acid-modified form of human Sonic hedgehog. J. Biol. Chem..

[102] Porter J.A., Ekker S.C., Park W.J., von Kessler D.P., Young K.E., Chen C.H., Ma Y., Woods A.S., Cotter R.J., Koonin E.V. (1996). Hedgehog patterning activity: Role of a lipophilic modification mediated by the carboxy-terminal autoprocessing domain. Cell.

[103] Chen M.H., Li Y.J., Kawakami T., Xu S.M., Chuang P.T. (2004). Palmitoylation is required for the production of a soluble multimeric Hedgehog protein complex and long-range signaling in vertebrates. Genes Dev..

[104] Zeng X., Goetz J.A., Suber L.M., Scott W.J., Schreiner C.M., Robbins D.J. (2001). A freely diffusible form of Sonic hedgehog mediates long-range signalling. Nature.

[105] Gallet A., Ruel L., Staccini-Lavenant L., Therond P.P. (2006). Cholesterol modification is necessary for controlled planar long-range activity of Hedgehog in Drosophila epithelia. Development.

[106] Goetz J.A., Singh S., Suber L.M., Kull F.J., Robbins D.J. (2006). A highly conserved amino-terminal region of sonic hedgehog is required for the formation of its freely diffusible multimeric form. J. Biol. Chem..

[107] Billings P.C., Pacifici M. (2015). Interactions of signaling proteins, growth factors and other proteins with heparan sulfate: Mechanisms and mysteries. Connect. Tissue Res..

[108] Radhakrishnan A., Sun L.P., Kwon H.J., Brown M.S., Goldstein J.L. (2004). Direct binding of cholesterol to the purified membrane region of SCAP: Mechanism for a sterol-sensing domain. Mol. Cell.

[109] Tukachinsky H., Kuzmickas R.P., Jao C.Y., Liu J., Salic A. (2012). Dispatched and scube mediate the efficient secretion of the cholesterol-modified hedgehog ligand. Cell Rep..

[110] Kawakami T., Kawcak T., Li Y.J., Zhang W., Hu Y., Chuang P.T. (2002). Mouse dispatched mutants fail to distribute hedgehog proteins and are defective in hedgehog signaling. Development.

[111] Jakobs P., Exner S., Schurmann S., Pickhinke U., Bandari S., Ortmann C., Kupich S., Schulz P., Hansen U., Seidler D.G. (2014). Scube2 enhances proteolytic Shh processing from the surface of Shh-producing cells. J. Cell Sci..

[112] Yasuda T., Mundy C., Kinumatsu T., Shibukawa Y., Shibutani T., Grobe K., Minugh-Purvis N., Pacifici M., Koyama E. (2010). Sulfotransferase Ndst1 is needed for mandibular and TMJ development. J. Dent. Res..

[113] Crawford B.E., Garner O.B., Bishop J.R., Zhang D.Y., Bush K.T., Nigam S.K., Esko J.D. (2010). Loss of the heparan sulfate sulfotransferase, Ndst1, in mammary epithelial cells selectively blocks lobuloalveolar development in mice. PLoS ONE.

[114] Grobe K., Inatani M., Pallerla S.R., Castagnola J., Yamaguchi Y., Esko J.D. (2005). Cerebral hypoplasia and craniofacial defects in mice lacking heparan sulfate Ndst1 gene function. Development.

[115] Long H.Q., Tian P.F., Guan Y.X., Liu L.X., Wu X.P., Li B. (2019). Expression of Ihh signaling pathway in condylar cartilage after bite-raising in adult rats. J. Mol. Histol..

[116] Kinumatsu T., Shibukawa Y., Yasuda T., Nagayama M., Yamada S., Serra R., Pacifici M., Koyama E. (2011). TMJ development and growth require primary cilia function. J. Dent. Res..

[117] Buckland J. (2010). Osteoarthritis: Blocking hedgehog signaling might have therapeutic potential in OA. Nat. Rev. Rheumatol..

